# *In-Vitro* Antagonistic Characteristics of Crude Aqueous and Methanolic Extracts of *Garcinia kola* (Heckel) Seeds against Some *Vibrio* Bacteria

**DOI:** 10.3390/molecules16042754

**Published:** 2011-03-24

**Authors:** Dambudzo Penduka, Omobola O. Okoh, Anthony I. Okoh

**Affiliations:** 1 Applied and Environmental Microbiology Research Group (AEMREG), Department of Biochemistry and Microbiology, University of Fort Hare, Alice, South Africa; E-Mail: 201007033@ufh.ac.za; 2 Department of Chemistry, University of Fort Hare, Alice, South Africa; E-Mail: ookoh@ufh.ac.za

**Keywords:** *Vibrio* species, *Garcinia kola*, methanol extract, aqueous extract, MIC, rate of kill

## Abstract

The methanolic and aqueous extracts of *Garcinia kola* seeds were screened for their anti-*Vibrio* activities against 50 *Vibrio* isolates obtained from wastewater final effluents in the Eastern Cape Province, South Africa. The crude extracts at 10 mg/mL exhibited appreciable inhibitory activities against most of the test *Vibrio* isolates, with zones of inhibition ranging from 10–19 mm for methanol extract and 8–15 mm for the aqueous extracts. The minimum inhibitory concentrations (MIC) of the methanol extract varied from 0.313 to 2.5 mg/mL while that for the aqueous extract was 10 mg/mL for all the susceptible *Vibrio* isolates. Rate of kill assay of the methanolic extracts against three selected Vibrio species showed bacteriostatic activities against all of them achieving 58% and 60% (*Vibrio vulnificus* AL042); 68% and 69% (*Vibrio parahaemolyticus* AL049);and 70% and 78% (*Vibrio fluvialis* AL040) killing of the test bacteria at 3× and 4 ×MIC values, respectively, after 2 h exposure time. We conclude that *Garcinia kola* seeds hold promise as a potential source of therapeutic compounds of relevance in *Vibrio* infections management.

## 1. Introduction

*Vibrio* species are ubiquitous in the aquatic environment. They appear at particularly high densities in and/or on marine organisms including corals, fish, molluscs, sea grass, sponges, shrimps and zooplankton [1]. Among the major diseases caused by *Vibrio* species is cholera, which occurs when *V. cholerae* colonizes the small intestine and releases an enterotoxin [2]. *V. parahaemolyticus, V. alginolyticus* and *V. vulnificus* are also known to cause seafood-borne infections such as septicemia and wound infections, and *V. vulnificus* has been reported to be responsible for 95% of seafood-related deaths [3].

Extra intestinal *Vibrio* infections often result in serious disability or death [4]. Infections by *V. vulnificus*, *V. parahaemolyticus*, and possibly *V. cholerae* non-01 are more likely to cause primary septicemia in persons with pre-existing liver disease such as chronic hepatitis, cirrhosis, iron-storage diseases and compromised immune system from conditions like chronic renal insufficiency, cancer, or diabetes [5]. 

*Vibrio* species are not an exception when it comes to antibiotic resistant strains. Several studies [6,7,8,9] have reported the emergence of such strains. The development of antibiotic resistance outpaces the development of new drugs such that it has become a worldwide problem with deleterious long-term effects [10]. In developing countries, factors such as inadequate access to effective drugs, unregulated dispensing and manufacture of antibiotics and truncated antibiotic therapy because of cost are contributing to the development of multi-drug resistant organisms [10].

Traditional medicines represented mainly by plants have become an alternative as they are considered relatively safer and more affordable when compared to synthetic antibiotics. Hence the need to increase the body of knowledge on the antimicrobial activities of some traditional medicinal plants such as *Garcinia kola* towards curbing the effects of antibiotic resistance in such virulent pathogens as *Vibrio* species becomes imperative. 

*Garcinia kola* is a plant of west and central African origin [11]. It is commonly referred to as bitter kola for its bitter taste and has the popular acronym “wonder plant” amongst the southwestern Nigerian people because every part of it has been found to be of medicinal importance [12]. In Nigeria, the seed is chewed for the relief of cough, colds, colic, hoarseness of voice, and throat infections. The plant is also used for the treatment of liver disorders, jaundice, high fever and as a purgative and chewing stick [11]. The seed has proven antimicrobial activities [13,14,15,16] and it has been employed in the treatment of various ailments.

The phytochemical analysis of a 3:2 methanol and sterile distilled water extract of *Garcinia kola* seed powder revealed the presence of flavonoids, tannins, cardiac glycosides, steroids, saponins and reducing sugars. These phytochemical compounds are known to play important roles in the bioactivity of medicinal plants [15]. Although studies have been carried out that show the antimicrobial activities of crude extracts of *Garcinia kola* seeds, to the best of our knowledge there is paucity of information of the anti*-Vibrio* potential of the aqueous and methanolic extracts of the seeds of this plant, especially against environmental strains of the bacteria such as those isolated from wastewater environments. In the light of the increasing trend of multiple antibiotic resistance in *Vibrio* species isolated in the South African aquatic milieu [9] and the pathogenicity of *Vibrio* species to humans [17] the exploration for new anti*-Vibrio* compounds especially of plants origin becomes necessary. In this paper therefore we report on the anti-*Vibrio* potentials of crude aqueous and methanol extracts of *Garcinia kola* seeds. 

## 2. Results and Discussion

### 2.1. Results

The results of the anti-*Vibrio* activities of the methanol and aqueous crude extracts of *Garcinia kola* seeds are shown in Table 1.The methanol extract showed activity against 16 (34%) of the test bacteria, whilst the aqueous extract had activity against 12 (24%) out of the 50 *Vibrio* isolates. The zones of inhibition ranged from 8–20 mm for methanol extracts and 8–14 mm for the aqueous extracts. *V. fluvialis* (AL040) had the highest zones of inhibition for both extracts. All the isolates that were susceptible to the aqueous extract were susceptible to the methanol extract as well. It appears that the methanol extract has more potent bacterial activity compared to the aqueous extract. The 5% DMSO and sterile distilled water negative controls had no anti-*Vibrio* activity on any of the tested *Vibrio* species.

The MIC and MBC results are presented in Table 2. The methanol extract had MIC and MBC values ranging from 0.313–2.5 mg/mL and 10– >10 mg/mL respectively, whilst for the aqueous extracts the MIC and MBC values were higher ranging from 10– >10 mg/mL and above 10 mg/mL respectively. *V. fluvialis* (AL031) and *V. parahaemolyticus* (AL032) had the lowest MIC values of 0.313 mg/mL for methanol and the highest MIC value was from *V. vulnificus* (AL042) (2.5 mg/mL). For the aqueous extract *V. vulnificus* (AL042) and *V. fluvialis* (AL022) had the highest MIC values of >10 mg/mL, whilst all the other *Vibrios* had MIC values of 10 mg/mL. The MBC values for the methanol extract were above 10 mg/mL for five *Vibrio* isolates whilst the rest of the isolates had values of 10 mg/mL, for aqueous extracts all the isolates had MBC values of above 10 mg/mL.

**Figure 1:**
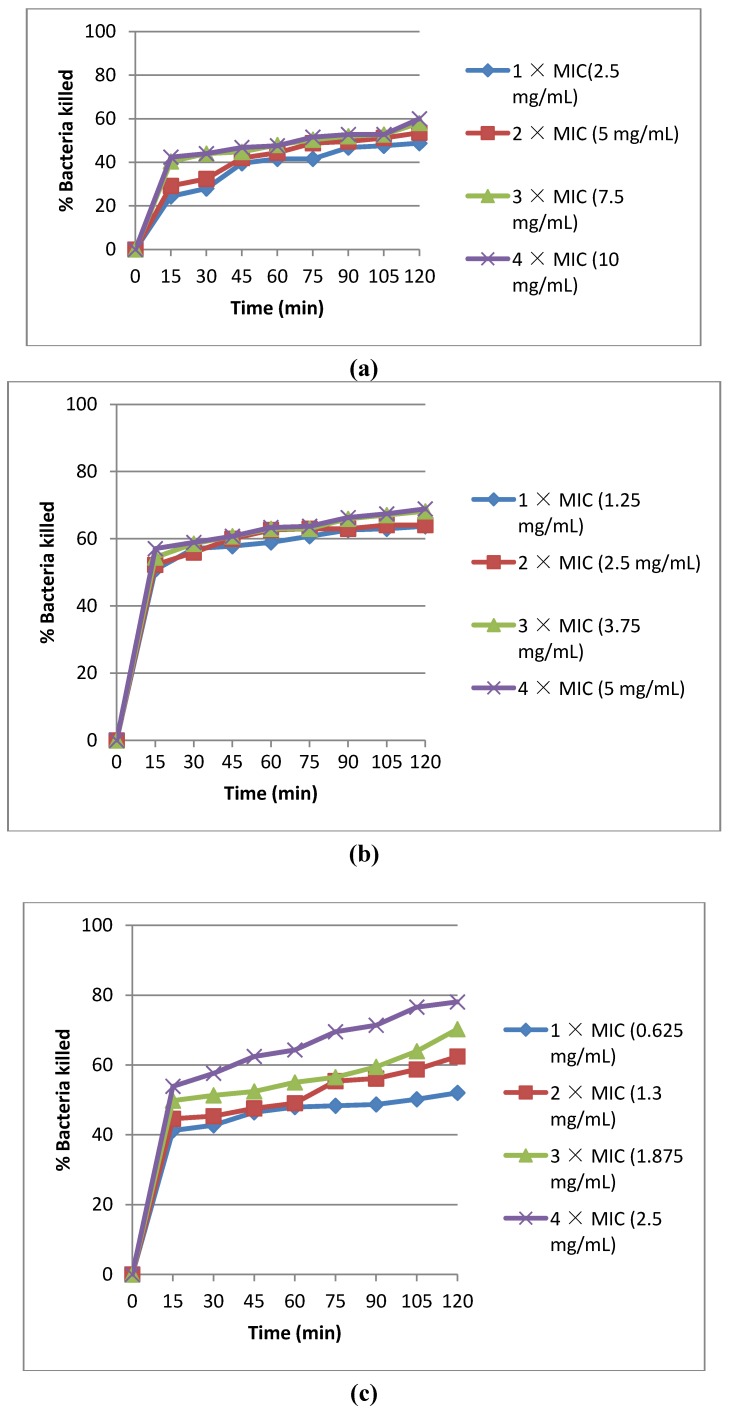
**(a)** Rate of kill of *V. vulnificus* (AL042) by crude methanol extract of *Garcinia kola* seeds. **(b)** Rate of kill of *V. parahaemolyticus* (AL049) by crude methanol extract of *Garcinia kola* seeds. **(c)** Rate of kill of *V. fluvialis* (AL040) by crude methanol extract of *Garcinia kola* seeds.

The rate of kill assay was carried out for the methanol extract only based on its higher activity compared to the aqueous extract. Three different *Vibrio* species namely *V. vulnificus* (AL042), *V. parahaemolyticus* (AL049) and *V. fluvialis* (AL040) were selected for this analysis and the results are as shown in Figure 1a, Figure 1b and Figure 1c respectively. The percentage of bacteria cells killed at 1×, 2×, 3× and 4 × MIC values respectively for each *Vibrio* specie after 2 hour exposure time, were 48.8, 53.6, 58 and 60% for *V. vulnificus* (AL042) (Figure 1a); 63.7, 64.1, 68.2 and 68.9% for *V. parahaemolyticus* (AL049) (Figure 1b); and 52.0, 62.5, 70.3 and 78.1 % for *V. fluvialis* (AL040) (Figure 1c). The number of bacteria cells killed for each *Vibrio* specie increased as the time and the concentration of the extract increased.

**Table 1:** Anti-*Vibrio* activities of Ciprofloxacin and the crude methanol and aqueous extracts of *Garcinia kola* seeds.

ORGANISM	M	A	C	ORGANISM	M	A	C
*Vibrio* species ( EL 031)	- (0)	- (0)	+(10)	*Vibrio* species (AL 020)	+ (15)	+ (8)	+(21)
*V. parahaemolyticus* (AL 043)	+ (15)	+ (8)	+(21)	*V. vulnificus* (AL 001)	- (0)	- (0)	+(20)
*V. fluvialis* (AL 025)	- (0)	- (0)	+(27)	*V. fluvialis* (AL002)	- (0)	- (0)	+(20)
*Vibrio* species (AL021)	+ (13)	- (0)	+(20)	*Vibrio* species (AL035)	- (0)	- (0)	+(20)
*V. vulnificus* (AL042)	+ (13)	+ (10)	+(20)	*V. vulnificus* (AL048)	+ (8)	- (0)	+(15)
*V. metschnikovii* (AL012)	- (0)	- (0)	+(26)	*V. vulnificus* (AL018)	- (0)	- (0)	+(21)
*V. vulnificus* (AL041)	- (0)	- (0)	+(21)	*V. fluvialis* (AL036)	- (0)	- (0)	+(13)
*Vibrio* species (AL 050)	- (0)	- (0)	+(23)	*V. fluvialis* (AL013)	- (0)	- (0)	+(26)
*V. fluvialis* (AL 022)	+ (12)	+ (9)	+(19)	*V. parahaemolyticus* (AL017)	- (0)	- (0)	+(26)
*V. vulnificus* (AL 024)	- (0)	- (0)	+(20)	*V. vulnificus* (AL038)	- (0)	- (0)	+(20)
*V. fluvialis* (AL014)	- (0)	- (0)	+(20)	*V. parahaemolyticus* (AL049)	+ (12)	+ (9)	+(19)
*V. parahaemolyticus* (AL009)	- (0)	- (0)	+(22)	*V. vulnificus* (AL011)	- (0)	- (0)	+(29)
*V. fluvialis* (AL037)	- (0)	- (0)	+(16)	*V. fluvialis* (AL033)	- (0)	- (0)	+(15)
*V. vulnificus* (AL039)	- (0)	- (0)	+(21)	*V. fluvialis* (AL004)	+ (11)	- (0)	+(26)
*V. parahaemolyticus* (DM 015)	- (0)	- (0)	+(25)	*V. parahaemolyticus* (AL003)	- (0)	- (0)	+(20)
*Vibrio* species (AL005)	- (0)	- (0)	+(16)	*V. fluvialis* (AL006)	- (0)	- (0)	+(27)
*V. fluvialis* (AL031)	+ (15)	+ (8)	+(20)	*V. fluvialis* (AL027)	- (0)	- (0)	+(27)
*V. fluvialis* (AL040)	+ (20)	+ (14)	+(20)	*Vibrio* species (EL 027)	- (0)	- (0)	+(27)
*V. parahaemolyticus* (AL008)	- (0)	- (0)	+(30)	*V. vulnificus* (AL015)	- (0)	- (0)	+(20)
*V. parahaemolyticus* (AL030)	+ (11)	+ (8)	+(23)	*V. parahaemolyticus* (AL032)	+ (13)	+ (8)	+(19)
*V. parahaemolyticus* (EL009)	+ (14)	- (0)	+(20)	*V. vulnificus* (AL044)	- (0)	- (0)	+(15)
*V. vulnificus* (AL029)	- (0)	- (0)	+(20)	*V. parahaemolyticus* (AL045)	+ (12)	+ (9)	+(20)
*V. metschnikovii* (AL023)	+ (12)	+ (8)	+(17)	*Vibrio* species (AL047)	- (0)	- (0)	+(27)
*V. fluvialis* (AL019)	+ (11)	+ (8)	+(19)	*V. metschnikovii* (AL 016)	- (0)	- (0)	+(20)
*V. parahaemolyticus* (AL028)	- (0)	- (0)	+(20)	*Vibrio* species (EL 047)	- (0)	- (0)	+(15)

Key: (+) denotes susceptible to the extract, (-) denotes not susceptible, (number) denotes diameter of zone of inhibition in mm, M denotes methanol extract, A denotes aqueous extract, C denotes Ciprofloxacin.

**Table 2:** Minimum inhibitory concentrations (MIC) and minimum bactericidal concentrations (MBC) of the methanol and aqueous extracts against the susceptible *Vibrio* isolates.

**ORGANISM**	**EXTRACTS**			
	**METHANOL**		**AQUEOUS**	
	MIC (mg/mL)	MBC (mg/mL)	MIC (mg/mL)	MBC (mg/mL)
*V. vulnificus* (AL042)	2.5	>10	>10	>10
*V. fluvialis* (AL019)	1.25	>10	10	>10
*V. parahaemolyticus* (AL049)	1.25	>10	10	>10
*V. parahaemolyticus* (AL045)	1.25	>10	10	>10
*Vibrio.* species (AL021)	0.625	10	-	-
*V. fluvialis* (AL022)	0.625	10	>10	>10
*V. metschnikovii* (AL023)	0.625	10	10	>10
*V. parahaemolyticus* (AL030)	0.625	10	10	>10
*Vibrio.* species (AL020)	0.625	10	10	>10
*V. fluvialis* (AL040)	0.625	10	10	>10
*V. fluvialis* (AL031)	0.313	10	10	>10
*V. parahaemolyticus* (AL032)	0.313	10	10	>10
*V. parahaemolyticus* (AL043)	0.625	10	10	>10
*V. parahaemolyticus* (EL009)	0.625	10	-	-
*V. fluvialis* (AL004)	0.625	10	-	-
*V. vulnificus* (AL048)	1.25	>10	-	-

Key: MIC denotes minimum inhibitory concentration, MBC denotes minimum bactericidal concentration, - denotes not determined.

### 2.2. Discussion

This study has revealed that both methanolic and aqueous extracts of the *Garcinia kola* seeds have antagonistic activities against *V. vulnificus*, *V. fluvialis*, *V. parahaemolyticus* and *V. metschnikovii* and some unidentified *Vibrio* species. The antagonistic activity exhibited by the aqueous extract validates the traditional use of the plant for the treatment of diarrhoea [12], high fever [11], stomach aches [18] and abdominal colicky pain [19], as some *Vibrio* species such as *V. parahaemolyticus* and *V. vulnificus* are food poisoning bacteria that can cause such symptoms [20]. Our finding also corroborates previous reports on the antibacterial activities of *Garcinia kola* seeds [13,14,16]. Nevertheless, Ogbulie *et al.* [21] reported findings contrary to ours. In that study, they showed that both hot and cold aqueous and ethanolic extracts of *Garcinia kola* seeds had no activity against a *Vibrio* specie used in the study, although they had variable activities against other Gram negative bacteria such as *Escherichia coli*, *Salmonella typhi* and *Pseudomonas aeruginosa*, with the ethanolic extracts having more activity compared to the aqueous extracts. The limitations of the study by Ogbulie *et al.* [21] in comparison to ours is that only one *Vibrio* isolate was tested, so it cannot be used to conclusively determine the anti-*Vibrio* potential of *Garcinia kola* seeds.

Although both the aqueous and methanol extract of the *Garcinia kola* seeds had anti-*Vibrio* activities, the methanol extract was more active. The difference in activity between the two solvent extracts has been attributed to a better solubility of the active agents; xanthones, benzophenones, and flavonoids especially biflavonoid type GB1 [22,23] in organic solvents than in water [13,21,24,25,26]. These phytochemical compounds have been known to play different roles in the antimicrobial potential of medicinal plants. For example, previous reports have demonstrated the anti-diarrhoeal activity of tannin- [27], flavonoid- [28], saponin- and reducing sugar-containing [29] plant extracts. The phytochemicals outlined above are also present in *Garcinia kola* seeds and might be responsible for the anti-*Vibrio* activities found in this study, more so as most *Vibrio* species are implicated in diarrhoea.

The rate of kill of the selected test *Vibrio* species by the methanol extract proved to be generally concentration and time dependent, with the rate of kill increasing with increasing concentration of the extract and times of exposures (Figure 1a, Figure 1b and Figure 1c). The pattern of activity also suggests that the extract is bacteriostatic against all the three *Vibrio* species tested at 1×, 2×, 3× and 4 × MIC, in line with the interpretation key of Pankey and Sabath [30] which recommended a 99.9% reduction in cell counts to be considered bactericidal. The percentages of cells killed during the rate of kill experiment varied from 48.8 to 78.1%, thus suggesting *Garcinia kola* to be a potential source of active compounds of relevance in anti-*Vibrio* chemotherapy.

## 3. Materials and Methods

### 3.1. Plant material

Ground powder of the *Garcinia kola* seeds were obtained from the plant material collection of the Applied and Environmental Microbiology Research Group (AEMREG) laboratory, University of Fort Hare Alice. South Africa. 

### 3.2. Preparation of extracts

The solvent extracts of the plant were prepared in accordance with the description of [31]. Briefly, the seed powder (100 g) was steeped in the respective solvent (methanol or water, 500 mL) for 48 h with shaking. The resultant extract was centrifuged at 3,000 rpm for 5 min at 4 °C. The supernatant was then filtered through Whatman No.1 filter paper, while the residue was then used in the second extraction with 300 mL of the respective solvent. After the second extraction process, the aqueous extract was freeze-dried at −50 °C under vacuum, whereas methanol extracts were concentrated under reduced pressure using a rotary evaporator at 65 °C. The concentrated extracts were then allowed to dry to a constant weight under a stream of air in a fume cupboard at room temperature. The methanol extracts were insoluble in water hence, dimethylsulphoxide (DMSO) at a concentration equal to 5% (v/v) was used to aid the reconstitution of the extracts when making different test concentrations whilst, the water extracts were reconstituted in sterile distilled water. 

### 3.3. Test Vibrio strains

The test *Vibrio* isolates (50 in all) used in this study were obtained from the culture collection of the Applied and Environmental Microbiology Research Group (AEMREG) laboratory at the University of Fort Hare, Alice, South Africa. The bacteria were isolated from wastewater effluents [9,32] and belonged to five species groups viz. *Vibrio*. sp. (unidentified to the species level), *V. parahaemolyticus*, *V. fluvialis*, V. *vulnificus*, *V. metschnikovii*. 

### 3.4. Preparation of the Inoculum

The inoculums of the test organisms were prepared using the colony suspension method [33]. Colonies picked from 24 hour old cultures grown on nutrient agar plates were used to make suspensions of the test organisms in saline solution (0.85% NaCl) to give an optical density of approximately 0.1 at 600 nm. The suspension was then diluted a hundred-fold before use.

### 3.5. Antibacterial susceptibility test

The sensitivity of each crude extract of the plant was determined using the agar well diffusion method as described by [34], with modifications. The prepared bacterial suspension (100 µL) was inoculated into sterile molten Mueller-Hinton agar medium at 50 °C in a MacCartney bottle, mixed gently and then poured into a sterile petri dish and allowed to solidify. A sterile 6 mm diameter cork borer was used to bore wells into the agar medium. The wells were then filled up with approximately 100 µL of the extract solution at a concentration of 10 mg/mL taking care to prevent spillage onto the surface of the agar medium. The plates were allowed to stand on the laboratory bench for 1 hour to allow proper diffusion of the extract into the medium after which the plates were incubated at 37 °C for 24 hours, and thereafter the plates were observed for zones of inhibition and measured. Ciprofloxacin (2 µg/mL) was used as a positive control, and distilled water was used as the negative control while 5% DMSO was also tested to determine its effect on each organism.

### 3.6. Determination of the minimum inhibitory concentration (MIC) and minimum bactericidal concentration (MBC)

The MICs were determined only for the test *Vibrio* that had shown susceptibility to the crude extracts using the broth microdilution method as outlined by [33] in sterile disposable flat-bottomed 96-well microtiter plates. Two-fold serial dilutions using sterile distilled water were carried out from 10 mg/mL stock plant extracts to make nine test concentrations ranging from 0.039 to 10 mg/mL for each solvent extract. A 100 µL volume of double strength Mueller-Hinton broth was introduced into all the 96 wells and 50 µL of the varying concentrations of the extracts were added in decreasing order along with 50 µL of the test organism suspension. Column 1 was used as the sterility wells containing 100 µL of the Mueller-Hinton broth and 100 µL sterile distilled water, column 2 was used as the positive control wells containing 100 µL of the broth, 50 µL of ciprofloxacin and 50 µL of the test organism whilst column 3 was used as the negative control wells containing 100 µL of the broth, 50 µL sterile distilled water and 50 µL of the test organism whilst columns 4 to 12 were used as test wells containing 100 µL of the broth, 50 µL of the extract concentration and 50 µL of the test *Vibrios*. The plates were then incubated at 37 °C for 18–24 h. Results were read visually by adding 40 µL of 0.2 mg/mL of *ρ*-iodonitrotetrazolium violet (INT) dissolved in sterile distilled water into each well [35]. A pinkish coloration is indicative of microbial growth because of their ability to convert INT to red formazan [36]. The MIC was recorded as the lowest concentration of the extract that prevented the appearance of visible growth of the organism after 24 hour of incubation [33].

The minimum bactericidal concentration (MBC) was determined from the MIC broth microdilution assays by subculturing 10 µL volumes from each well that did not exhibit growth after 24 hours of incubation and spot inoculating it onto fresh Mueller-Hinton agar plates [37]. The plates were incubated for 48 hours after which the number of colonies were counted. The MBC was defined as the lowest concentration killing more than or equal to 99.9% of the inoculum compared with initial viable counts [37].

### 3.7. Rate of kill assay

The time kill assay was done according to the method of [38]. Three selected test *Vibrio* isolates namely *V. vulnificus* (AL042), *V. parahaemolyticus* (AL049) and *V. fluvialis* (AL040) were used for the rate of kill studies on the basis of grouping on MIC levels viz 0.625, 1.25 and 2.5 mg/mL and medical importance of the species. The assay was done for the methanol extract only which proved to be more active when compared to the aqueous extract. The turbidity of the 18 hour old test *Vibrio* was first standardized to 10^8^ cfu/mL. Four different concentrations of the plant extract were made starting from the MIC to 4 × MIC value for each test organism. A volume (0.5 mL) of known cell density from each organism suspension was added to different concentrations of the extract solutions (4.5 mL), held at room temperature and the rate of kill determined over a period of 2 hours. Exactly 0.5 mL of each suspension was withdrawn at 15 minutes intervals and transferred to nutrient broth recovery medium containing 3% Tween 80 (4.5 mL) to neutralize the effects of the antimicrobial compound carryovers on the test organisms [15]. The suspension was then serially diluted and 0.5 mL was plated out for viable counts using the pour plate method. The plates were thereafter incubated at 37 °C for 48 hours. The control plates contained the test organism without the plant extracts. The emergent colonies were counted and compared with the counts of the culture control. 

## 4. Conclusions

This study has shown that both aqueous and methanol extracts of the seeds of *Garcinia kola* have antagonistic activities against *Vibrio* species, although the methanol extract appears to be more active. The low MIC values observed for the methanol extract are good starting points for further research that can lead to the isolation, purification and characterization of active compounds for new anti-*Vibrio* drug development purposes, which is a subject of on-going investigation in our group. 
